# Prostate biopsy techniques and pre-biopsy prophylactic measures: variation in current practice patterns in the Netherlands

**DOI:** 10.1186/s12894-020-00592-8

**Published:** 2020-03-12

**Authors:** Sofie C. M. Tops, Evert L. Koldewijn, Diederik M. Somford, Anita M. P. Huis, Eva Kolwijck, Heiman F. L. Wertheim, Marlies E. J. L. Hulscher, J. P. Michiel Sedelaar

**Affiliations:** 1 Department of Medical Microbiology, Radboud Center Infectious Diseases, Radboudumc, huispost 777, Geert Grooteplein Zuid 10, 6525 GA Nijmegen, the Netherlands; 2 Department of Urology, Catharina Hospital, Eindhoven, the Netherlands; 3 Department of Urology, Canisius-Wilhelmina Hospital, Nijmegen, the Netherlands; 4 Scientific Center for Quality of Healthcare, Radboud Institute for Health Sciences, Radboudumc, Nijmegen, the Netherlands; 5 Department of Urology, Radboudumc, Nijmegen, the Netherlands

**Keywords:** Antibiotic prophylaxis, Antibiotic resistance, Healthcare research, Prostate biopsy, Transrectal biopsy

## Abstract

**Background:**

The clinical landscape of prostate biopsy (PB) is evolving with changes in procedures and techniques. Moreover, antibiotic resistance is increasing and influences the efficacy of pre-biopsy prophylactic regimens. Therefore, increasing antibiotic resistance may impact on clinical care, which probably results in differences between hospitals. The objective of our study is to determine the (variability in) current practices of PB in the Netherlands and to gain insight into Dutch urologists’ perceptions of fluoroquinolone resistance and biopsy related infections.

**Methods:**

An online questionnaire was prepared using SurveyMonkey® platform and distributed to all 420 members of the Dutch Association of Urology, who work in 81 Dutch hospitals. Information about PB techniques and periprocedural antimicrobial prophylaxis was collected. Urologists’ perceptions regarding pre-biopsy antibiotic prophylaxis in an era of antibiotic resistance was assessed. Descriptive statistical analysis was performed.

**Results:**

One hundred sixty-one responses (38.3%) were analyzed representing 65 (80.3%) of all Dutch hospitals performing PB. Transrectal ultrasound guided prostate biopsy (TRUSPB) was performed in 64 (98.5%) hospitals. 43.1% of the hospitals (also) used other image-guided biopsy techniques. Twenty-three different empirical prophylactic regimens were reported among the hospitals. Ciprofloxacin was most commonly prescribed (84.4%). The duration ranged from one pre-biopsy dose (59.4%) to 5 days extended prophylaxis. 25.2% of the urologists experienced ciprofloxacin resistance as a current problem in the prevention of biopsy related infections and 73.6% as a future problem.

**Conclusions:**

There is a wide variation in practice patterns among Dutch urologists. TRUSPB is the most commonly used biopsy technique, but other image-guided biopsy techniques are increasingly used. Antimicrobial prophylaxis is not standardized and prolonged prophylaxis is common. The wide variation in practice patterns and lack of standardization underlines the need for evidence-based recommendations to guide urologists in choosing appropriate antimicrobial prophylaxis for PB in the context of increasing antibiotic resistance.

## Background

In the Netherlands, prostate biopsy (PB) is performed in approximately 40,000 patients every year [1]. Biopsies are performed to diagnose prostate cancer and form the basis for further staging patients with prostate cancer in either low, intermediate or high risk prostate cancer groups. Random systematic transrectal ultrasound guided prostate biopsy (TRUSPB) has been the preferred method for years. The field of PB, however, is undergoing rapid, significant change. Very recently, the European Association of Urology (EAU) guideline on prostate cancer has been updated [2]. An important modification in this guideline is the recommendation to also perform multiparametric magnetic resonance imaging (mpMRI) before PB in biopsy-naïve patients and, when mpMRI is positive (i.e. PI-RADS ≥3), to combine targeted and systematic biopsies. In addition, several more or less equivalent techniques are introduced to improve targeted biopsies [3]. These developments may have significant impact on the urological care provided in daily clinical practice, whereby it seems plausible that diagnostic differences exist between hospitals.

Moreover, (inter) national urological guidelines are unclear and lack standardization with regard to the recommended periprocedural prophylactic measures in PB [2, 4–7]. In Table 1 an overview of these guidelines is given [2, 4–7]. Guidelines strongly recommend the use of antimicrobial prophylaxis, generally with fluoroquinolones (FQ). The EAU guidelines on urological infections, however, states that the choice of regimen remains debatable [5]. An important reason for this is the increase in FQ resistant faecal flora, ranging from 10.6 to 92% between different countries, and 22% in the Netherlands, which is a potential threat for patients undergoing transrectal PB [8, 9].

**Table 1:** Overview of guidelines on the prevention of infectious complications after transrectal prostate biopsy

***Recommendations***	EAU guidelines on prostate cancer [2]	EAU guidelines on urological infections [5]	AUA guidelines [4]	Dutch guidelines [6, 7]
*First choice antimicrobial prophylaxis*	Fluoroquinolones with ciprofloxacin being superior to ofloxacin	Debatable, most commonly FQ are applied	Fluoroquinolones *or* 1st/2nd/3rd gen. Cephalosporin	Fluoroquinolones
*Alternative antimicrobial prophylaxis*	Not mentioned	Fosfomycin trometamol^a^	Co-trimoxazole *or* Aminoglycoside (Aztreonam)	If recent culture shows FQ resistance: antibiotics based on the resistance pattern, preferably co-trimoxazole
*Duration of prophylaxis*	Not mentioned	Debatable, meta-analysis by the guideline panel ongoing	≤ 24 h	Single dose
*Timing of prophylaxis*	Not mentioned	Not mentioned	≥ 1 h before prostate biopsy	Not mentioned
*Culture-guided prophylaxis*	Regional and local resistance patterns should be taken into account when deciding on the choice of antibiotics	Mentioned^a^	Not mentioned	There is no indication to routinely perform a urine or rectum culture
*Topical preparation*	Rectal disinfection with povidone-iodine may be considered.	Use rectal cleansing with povidone-iodine	No standard for topical preparation has been established	Not mentioned

^a^No strong recommendations are made

In the guidelines, there is no cut-off point in the level of resistance at which FQ would be deemed ineffective and no recommendations about what equally effective other prophylactic regimens could be used [2, 4–7]. Therefore, urologists must assess the risk of carrying FQ resistant faecal flora and consider alternatives for each patient individually, which affects daily care and leads to variation in prostatic biopsy practices.

There are already signs that differences in PB care exist between health care providers. Studies from various countries (Sweden, Germany, Australia and New Zealand, Ireland and the United States) showed a lack of uniformity in PB techniques and pre-biopsy prophylaxis [10–15].

The objective of our study is to gain insight into current Dutch prostatic biopsy practices and to determine the variation in practice patterns among Dutch urologists in the context of the risk of post-biopsy infections. In addition urologists’ perceptions of FQ resistance and biopsy related infections is assessed. It should be noted that at the time of our survey, the EAU guideline update was not published yet [2]. At that moment, guidelines stated to only perform mpMRI before repeat biopsy to allow targeted biopsies of suspicious lesions in addition to standard biopsies [4, 16].

## Methods

### Design and study population

In this observational study, a 5 min online questionnaire was prepared using SurveyMonkey® platform. Pilot testing was performed by members of the research team. Thereafter, the online questionnaire was distributed by the Dutch Association of Urology (DUA) to all its members. From the 81 Dutch hospitals, a total of 420 urologists and residents were approached to gain insight into the practices in their hospital. In April 2018, all DUA-members received an e-mail containing a unique link to complete the questionnaire. A reminder was sent after 2 weeks. Respondents were excluded from analysis if they only answered demographic questions or if the hospital of employment was unknown. Urologists from the Netherlands Antilles were also excluded from analysis.

All participants were informed about the voluntary nature of their participation and that their responses were kept confidential. They were able to access the questionnaire multiple times to allow for possible changes and completion at later times. No financial compensation was given in exchange for participation.

### Survey

The survey consisted of three parts (25 items), and mainly multiple-choice questions were used. In the first part (five items) general information was collected regarding gender, position, number of years working in the current position, hospital name and location.

The second part consisted of 12 questions about biopsy technique and pre-prostate biopsy patient preparation. More specifically, urologists were asked about the route of PB, type(s) of procedural imaging guidance, number of biopsy cores, whether prophylactic antibiotics were used, the name(s), route(s) of administration, dosage(s), timing and duration of antibiotics, prophylactic strategy (empirical or culture-guided) and whether other prophylactic measures were applied.

In the third part (eight items), respondents’ perceptions of FQ resistance and infectious complications in PB was assessed. Unfortunately, the answers to two questions were not interpretable due to methodological shortcomings. The full questionnaire is available in Supplementary Appendix I.

### Statistical analysis

Descriptive statistical analysis was performed using SPSS version 25.0 (IBM Corp., Armonk, NY, USA). Both completed and partially completed questionnaires were analyzed using the number of completed responses per item as the denominator.

To describe Dutch PB practice, answers to questions on biopsy technique and pre-biopsy patient preparation (part 2) were analyzed on hospital level, i.e. answers of different professionals from the same hospitals were counted once. In case of discrepancies in the answers of urologists from the same hospital, the urology department of the concerning hospital was contacted to reach consensus.

All answers to questions on characteristics of the study population and professionals perceptions on biopsy related infections (part 1 and 3) were analyzed on individual level, i.e. answers of different professionals from the same hospitals were counted separately.

## Results

Demographic characteristics are shown in Table 2. A total of 176 responses were received. Twelve respondents that only answered the demographic questions and three respondents from the Netherlands Antilles were excluded from the analysis. Questionnaires of 161 professionals (38.3%) were included in the final analyses, providing data on 65 (80.3%) Dutch hospitals [17]. Three of the professionals only partially answered the other questions. There were 41 (50.6%) hospitals where more than one urologist completed the questionnaire.

**Table 2:** Demographic characteristics

*Parameter*	*n (%)*
**Function**	
Urologist	141 (87.6)
Residents	20 (12.4)
**Sex**	
Male	106 (65.8)
Female	55 (34.2)
**Number of years working in the current position**	
0–5 years	37 (23.0)
5–10 years	52 (32.3)
≥ 10 years	72 (44.7)
**Working at**	
Academic hospital	24 (14.9)
Peripheral hospital or other institution	136 (84.5)
Unknown	1 (0.6)

### PB techniques

**Fig. 1:**
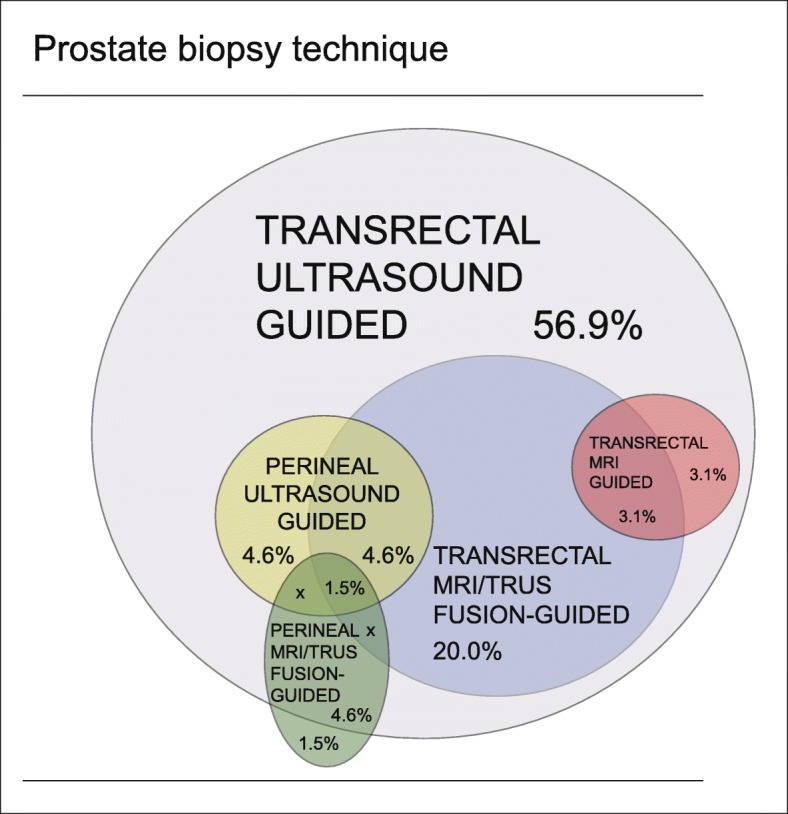
Variation in combinations of prostate biopsy techniques in the different hospitals. *Certain combinations do not exist, these are indicated with X*

There was a wide variation in combinations of applied PB techniques between Dutch hospitals: in total ten different combinations were used (Fig. 1). Schematic TRUSPB was performed in 64 (98.5%) hospitals. TRUSPB was the only method to obtain prostate tissue in 37 (56.9%) hospitals and was the most common procedure in 21 of the 27 hospitals with additional PB techniques. In four (6.2%) hospitals, transrectal MRI/TRUS fusion-guided PB was the most used method. Perineal MRI/TRUS fusion-guided PB and transrectal MRI guided PB was most frequently applied in respectively two (3.1%) and one (1.5%) hospital(s). Digitally directed PB was occasionally used in 12 (18.5%) hospitals.

In 57 of the 58 hospitals (98.5%) where TRUSPB was the most common procedure, urologists routinely obtained 8–12 cores per biopsy session. In one (1.5%) hospital, the number of biopsy cores during TRUSPB amounted to 12–16. In case MRI/TRUS fusion-guided or MRI guided PB was the most common method, there was a wide variation in the number of biopsy cores between hospitals. In four (57.1%) hospitals, targeted biopsies of mpMRI suspicious lesions were taken in addition to systematic biopsies (12–14 cores per biopsy session) and in three (42.9%) hospitals targeted biopsies were taken alone (2–6 cores per biopsy session).

We received one or more responses from all eight academic hospitals in the Netherlands. With regard to PB techniques, almost all academic hospitals also used methods other than TRUSB (87.5%). In two academic hospitals MRI/TRUS fusion-guided PB and in one academic hospital MRI guided PB was the most used method.

### Perioperative antimicrobial prophylaxis

**Fig. 2:**
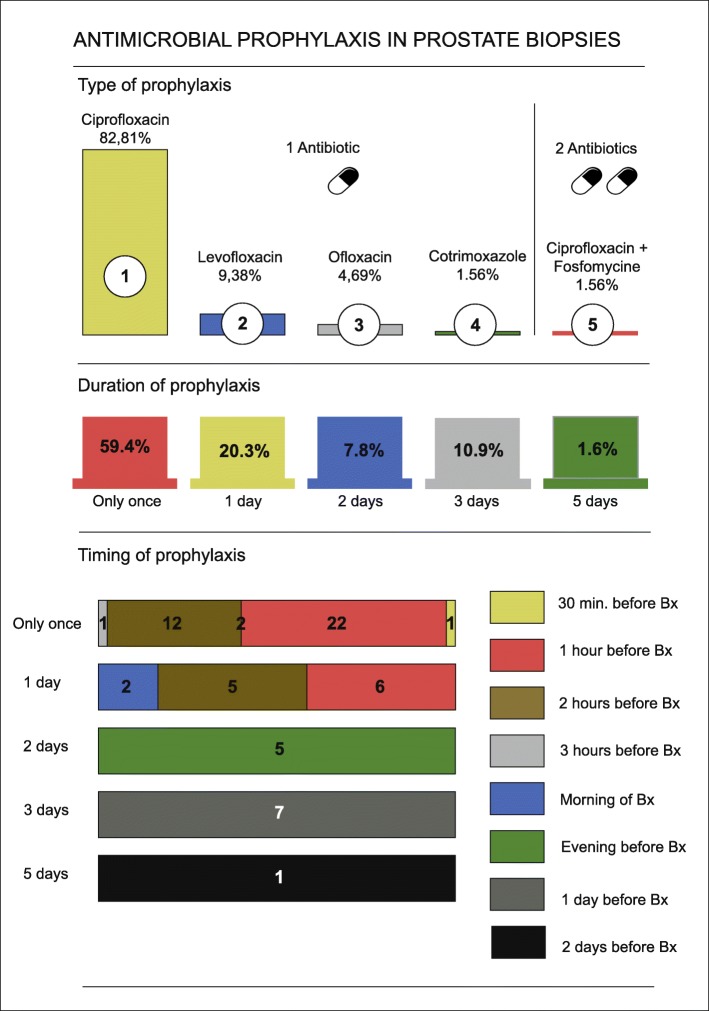
Variation in antimicrobial prophylaxis in prostate biopsies

Oral antimicrobial prophylaxis for transrectal PB was prescribed in all hospitals. There was a wide inter-hospital variation in antibiotic choice, duration and timing combinations: 23 different antibiotic regimens with five different antibiotics were reported. The antibiotics most frequently used were FQ, either alone (98.5%) or combined with another antibiotic (1.6%) (Fig. 2). In case (a combination with) ciprofloxacin was used (84.4%), a single dose consisted of 500 mg (90.7%), 750 mg (3.7%) or 1000 mg (5.6%). The duration of the prophylaxis ranged from one pre-biopsy dose to 5 days extended prophylaxis with most hospitals (59.4%) giving a single dose of antibiotics 1 hour prior to PB (Fig. 2).

In all hospitals, antimicrobial prophylaxis was given empirically according to a standardized protocol. Urologists from four hospitals (6.3%) reported to use rectal swab cultures prior to PB in patients at risk for FQ resistance. Other measures reported to prevent infectious complications after PB were screening for urinary tract infection by urine sediment and/or urine culture (3.1%), washing the biopsy needle with povidone-iodine solution (1.6%) and the use of laxative suppositories (3.1%). A rectal enema was included in the preparation in one hospital (1.6%).

With regard to antimicrobial prophylaxis, no differences between academic and peripheral hospitals were detected nor were there any differences according to regions. The antimicrobial prophylaxis regimens as specified by the respondents on hospital and individual level are described in Supplementary Appendix II.

### Urologists’ perception regarding antimicrobial prophylaxis in PB

According to 89 respondents (57.4%) the degree of infectious complications after PB in their hospital was stable, while 17 respondents (11.0%) indicated that they (subjectively) observed a decreasing number and 28 respondents (18.1%) an increasing number of infectious complications after PB. The other 21 respondents (13.5%) indicated that they did not know the trend of infectious complications after PB in their hospital.

After stating in our questionnaire that in urological patients the percentage of FQ resistant Gram-negative bacteria has increased from 7% in 2000 to 19% in 2017, we asked professionals if they were aware of this increasing level of FQ resistance [8]. Except for one respondent, all respondents (99.4%) indicated to be aware of the increasing FQ resistance. However, the degree of FQ resistance was assumed to be lower by 52 respondents (33.6%) and to be higher by four respondents (2.6%).

**Fig. 3:**
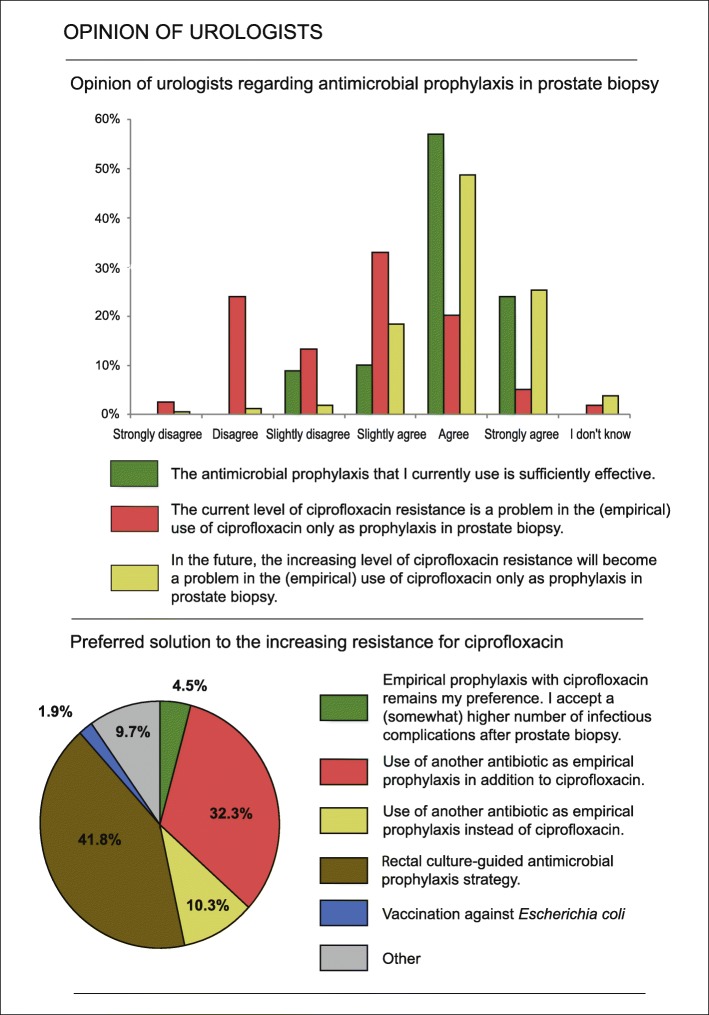
Urologists’ perception of antimicrobial prophylaxis in prostate biopsy

A total of 39 respondents (25.2%) (strongly) agreed with the statement that FQ resistance currently poses a problem in the empirical use of ciprofloxacin alone as prophylaxis around PB. One hundred fourteen respondents (73.6%) anticipate future problems in this area (Fig. 3). The preferred solutions of urologists to the increasing resistance for FQ can be found in Fig. 3. Besides the solutions provided in the questionnaire, urologists also suggested several solutions in the category *Other*. The most often mentioned ‘other’ solutions were: ‘if necessary, adapt antimicrobial prophylaxis based on local resistance data in consultation with the medical microbiologist or based on evidence-based guidelines’ (3.9%) and ‘perform more transperineal biopsies’ (3.2%).

## Discussion

This online survey among Dutch urologists showed a wide variation between hospitals both in PB techniques and in antimicrobial prophylaxis regimens.

Our survey showed that systematic TRUSPB with 8–12 cores per biopsy session was most frequently used (89.3%), which was in line with the guidelines at that moment [18]. Although our survey was performed before the recently published update of the EAU guidelines, urologists from 41.5% of the hospitals reported that they already perform MRI guided or MRI/TRUS fusion guided PB [2]. It suggests that (some) urologists, prior to guideline updates, already adjusted their practice patterns based on recent literature [19], which contributes to variation in daily care. In the Netherlands, saturation biopsies are not commonly performed.

Antimicrobial prophylaxis for PB is still dominated by ciprofloxacin monotherapy (84.4%), reflecting the recommendations in urological guidelines. However, given the rising prevalence of FQ resistance among relevant pathogens, antimicrobial prophylaxis with ciprofloxacin monotherapy is an increasingly doubtful policy. Many Dutch urologists are aware of the increased risk of FQ resistance and about a quarter of all urologists acknowledge rising FQ resistance as a current problem when prescribing antimicrobial prophylaxis. At this moment, only a small number of the Dutch hospitals are opting for a non-FQ based antibiotic regimen or have taken additional measures to address the problem of FQ resistance. We expect, however, that in the (near) future more Dutch hospitals will take measures trying to address rising FQ resistance, since 73.6% of all urologists anticipate future problems with regard to ciprofloxacin resistance and only 4.5% responded that ciprofloxacin remains their preference accepting a higher risk of urosepsis.

Furthermore, according to current guidelines the length of antimicrobial prophylaxis should be limited to < 24 h, which is not followed by 20% of the centers [4, 6, 7]. This deviation from the guidelines is in line with a study of Branch-Elliman et al. in which surgical antimicrobial prophylaxis was continued for > 24 h in 26.9% of all patients [20].

It is known that there is a wide variation in antimicrobial prophylaxis regimens internationally and between centers. Prior surveys on pre-prostate biopsy prophylaxis published since 2010 can be found in Supplementary Appendix III [10–15].

Variation in antibiotic choice could be related to a lack of strong evidence, which results in inconsistent and unclear recommendations in urological guidelines [2, 4–7]. While these guidelines generally recommend antimicrobial prophylaxis in transrectal PB, the choice of regimen, duration and the starting moment are ambiguous.

Moreover, the EAU guidelines leave individual clinicians to determine which patients may benefit from culture-guided prophylaxis [5]. It should be noted that although a recent meta-analysis [21] provided evidence for the use of culture-guided antimicrobial prophylaxis in reducing infectious complications after transrectal PB, a large prospective multicenter trial evaluating the effectiveness of rectal swab culture-guided antimicrobial prophylaxis, cost-effectiveness and optimal implementation is missing, but is currently being performed (NCT03228108).

Finally, the EAU guidelines recommend to use rectal cleansing with povidone-iodine prior to transrectal PB [5]. This is based on a meta-analysis of six trials including 1373 men that showed that the use of pre-biopsy rectal povidone-iodine preparation in addition to antimicrobial prophylaxis resulted in lower rates of infectious complications [0.58 (0.43 to 0.76); RR (95% CI)] [22–27]. However, not a single Dutch urologist reported to use rectal povidone-iodine cleansing prior to PB.

There is no cut-and-dried solution to the increasing resistance against FQ in the context of the risk of post-biopsy infection, which is supported by our finding that Dutch urologists have various preferred solutions to this problem. Although, in our survey, we asked urologists mainly for solutions with in the field of antimicrobial prophylaxis, it is important to note that the solution does not have to be (solely) based on the use of adapted or rectal culture-guided antimicrobial prophylaxis. Various studies found favourable infection rates for transperineal PB compared to TRUSB with similar rates of prostate cancer detection [2, 28–31]. Therefore, transperineal PB is an obvious alternative biopsy approach to avoid infections from FQ resistant bacteria. Our survey showed that transperineal biopsies were performed to some extent in 16.9% of all Dutch hospitals. In the Netherlands, transperineal biopsy is slow to be implemented in the clinic because of the consequent logistic problems such as the necessity for specialized staff and equipment, MRI capacity, greater labor time, higher costs etc. Moreover, mpMRI is likely to play an increasingly prominent role in the diagnostic pathway of prostate cancer. In the future, mpMRI could possibly reduce the number of biopsy cores and thereby possibly lower (infectious) complication rates without compromising detection rates or avoid PB in selected patients with negative mpMRI (index lesion PI-RADS ≤2) [32, 33].

The main strength of our study is that due to the high response rate (38.3% covering 80.2% of all Dutch hospitals) we were able to get a nationwide impression regarding the current Dutch prostatic biopsy practices.

The most important limitation of our study is that most of the results were analyzed at hospital level in order to obtain a representative picture of the variation in PB practice patterns between Dutch hospitals. Unfortunately, in hospitals where more than one urologist completed the questionnaire (50.6%), differences were also found between these intra-hospital urologists with regard to PB technique (41.5%) and antimicrobial prophylaxis (48.8%). However, in all cases the local protocol could be clarified. These intra-hospital differences again emphasize the lack of standardization in PB techniques and antimicrobial prophylaxis regimens in the Netherlands even among urologists who work in the same center. On individual level, 41 different antimicrobial prophylaxis regimens were reported instead of the 23 regimens we identified at hospital level (Supplementary Appendix II). In daily clinical practice, the variation is therefore probably even wider than the variation described in this article. Another limitation is that between the performance of our survey and publication of the results, the EAU guideline on prostate cancer [2] has been updated. Therefore, this survey does no longer depict the current international guidelines regarding prostate biopsy technique. This is, however, illustrative for the rapid evolutions in this area. Moreover, two questions were difficult to analyze due to methodological shortcomings and were left out. However, these questions were not critical for our analysis.

## Conclusions

The diversity of the reported prophylactic regimens is likely due to a lack of good evidence. Therefore, there is need for well designed, adequately powered trials of good quality in the field of urological pre-biopsy prophylactic measures. High quality research will result in evidence-based, clear-cut recommendations and offers urologists more guidance regarding antimicrobial prophylaxis around PB in the context of increasing antibiotic resistance. Professionals in daily practice have a need for dynamic, regularly updated guidelines that address changes in procedures, antimicrobial resistance patterns or new scientific insights. This study shows that the lack of standardized, clear guidelines lead to (undesirable) practice variation between health care professionals, which implies that patients in one hospital may receive better care than in another hospital. Moreover, this study shows that in an evolving clinical landscape of PB with changes in procedures, techniques and antimicrobial resistance, current urological guidelines lead to urologists making their own policies, resulting in an over-prescription of prophylactic antibiotics. This is ironically exactly what these guidelines aim to prevent.

## Supplementary information

**Additional file 1 Supplementary appendix I** Full questionaire. *The full online questionnaire that was distributed by the Dutch Association of Urology (DUA) to all its members.*

**Additional file 2 Supplementary file II** Antimicrobial prophylaxis regimens on hospital level and as specified by the (individual) respondents. *The antimicrobial prophylaxis regimens as specified by the respondents on hospital and individual level are described.*

**Additional file 3 Supplementary file III** Prior surveys on pre-prostate biopsy prophylaxis published since 2010. *Schematic overview of surveys conducted by others since 2010 about prophylactic measures around transrectal prostate biopsy*.

## Data Availability

The datasets used and/or analysed during the current study are available from the corresponding author on reasonable request.

## Abbreviations

DUA: Dutch Association of Urology
EAU: European Association of Urology
FQ: Fluoroquinolones
mpMRI: Multiparametric magnetic resonance imaging
PB: Prostate biopsy
TRUSPB: Transrectal ultrasound guided prostate biopsy
ZonMw: The Netherlands Organisation for Health Research and Development
