# Supplementary material for: Prostate biopsy techniques and pre-biopsy prophylactic measures: variation in current practice patterns in the Netherlands
Source: BMC Urol. 2020 Mar 12;20:24. doi: 10.1186/s12894-020-00592-8 (PMC7066741; doi:10.1186/s12894-020-00592-8)
Supplement: Supplementary file 1 — Additional file 1 Supplementary appendix I Full questionaire. The full online questionnaire that was distributed by the Dutch Association of Urology (DUA) to all its members. [file 12894_2020_592_MOESM1_ESM.docx]

**Supplementary Appendix I**

***Full questionnaire***

**Part 1**

**General Demographic Questions**

1. What is your gender?

- Male
- Female

1. What is your position?

- Urologist
- Resident in Urology
- Intern in Urology
- Physician Assistant
- Clinical nurse specialist

1. How long have you been working in this position?

**50 years**

**0 years**

1. At which institution are you employed?
2. At which location is the institution where you are employed?

**Part 2**

**Prostate biopsy**

1. What is the most used method of performing a prostate biopsy in the institution where you are employed?

In one of the following questions we will ask you about any other method(s) to perform prostate biopsy used in the institution where you are employed.

- Ultrasound-guided, perineal
- Ultrasound-guided, transrectal
- MRI-TRUS fusion-guided, perineal
- MRI-TRUS fusion-guided, transrectal
- MRI-guided, transrectal
- Digital-guided, transrectal
- No other method(s)
- Other (give further explanation)

1. What is the average number of core biopsies taken when using this method?
2. In the institution where you are employed, in how many percent of the cases this method is used to perform prostate biopsy?

If you have no insight into this, please enter “*I do not know”*.

1. Which other method(s) to perform prostate biopsy are used in the institution where you are employed?

- Ultrasound-guided, perineal
- Ultrasound-guided, transrectal
- MRI-TRUS fusion-guided, perineal
- MRI-TRUS fusion-guided, transrectal
- MRI-guided, transrectal
- Digital-guided, transrectal
- No other method(s)
- Other (give further explanation)

1. Is an enema administered to the patient prior to prostate biopsy in the institution where you are employed in order to reduce the risk of infectious complications?
   - No
   - Yes
   - Don’t know

Explanation:

1. Is antimicrobial prophylaxis given to the patient around prostate biopsy in the institution where you are employed in order to reduce the risk of infectious complications?
   - No
   - Yes
   - Don’t know

Explanation:

1. Are there other interventions (than enema or antimicrobial prophylaxis) applied in the institution where you are employed in order to prevent infectious complications after prostate biopsy?
   - No
   - Yes
   - Don’t know

Explanation:

**Antimicrobial prophylaxis for prostate biopsy**

1. How is the choice for the type of antimicrobial prophylaxis made in the institution where you are employed?
   - Empirical, according to local protocol
   - Empirical, at the discretion of the urologist
   - Culture-guided, order of preference for antimicrobial prophylaxis noted in local protocol
   - Culture-guided, at the discretion of the urologist or medical microbiologist
2. How many hours prior to prostate biopsy should patients take the antimicrobial prophylaxis according to the instructions?

**Optional, in case empirical antimicrobial prophylaxis is given (questions 13).**

1. How many antimicrobial agents are usually simultanously prescribed as prophylaxis around prostate biopsy in the institution where you are employed?
   - 1
   - 2
   - 3
2. Which antimicrobial agents are normally presribed as prophylaxis around prostate biopsy in the institution where you are employed?

**Antimicrobial agent 1:**

Name:

Dose (mg):

Duration (days):

Route of administration:

1. Please only answer this question, if you answered in question 15 that usually more than one antimicrobial agent is prescribed simultanously as prophylaxis around prostate biopsy in the institution where you are employed.

**Antimicrobial agent 2:**

Name:

Dose (mg):

Duration (days):

Route of administration:

**Antimicrobial agent 3:**

Name:

Dose (mg):

Duration (days):

Route of administration:

**Optional, in case culture-guided prophylaxis is given (questions 13).**

1. What is the antimicrobial agent of preference as prophylaxis around prostate biopsy (if case of culture-proven susceptibility) in the institution where you are employed?

Name:

Dose (mg):

Duration (days):

Route of administration:

1. Which alternative antimicrobial agents are prescribed as prophylaxis in case of resistancy for the antimicrobial agent of preference (based on rectal culture)?

**Alternative 1:**

Name:

Dose (mg):

Duration (days):

Route of administration:

1. **Alternative 2:**

Name:

Dose (mg):

Duration (days):

Route of administration:

**Part 3**

**Your opinion regarding antimicrobial prophylaxis**

1. In your opinion, is the antimicrobial prophylaxis that you currently use for prostate biopsy is sufficiently effective?

- Strongly disagree
- Disagree
- Slightly disagree
- Slightly agree
- Agree
- Strongly agree
- Don’t know

1. In your opinion, is the current level of ciprofloxacin resistance a problem in the (empiral) use of ciprofloxacin only as prophylaxis in prostate biopsy?

- Strongly disagree
- Disagree
- Slightly disagree
- Slightly agree
- Agree
- Strongly agree
- Don’t know

1. In your opinion, in the future, will the increasing level of ciprofloxacin resistance become a problem in the (empirical) use of ciprofloxacin only as prophylaxis in prostate biopsy?

- Strongly disagree
- Disagree
- Slightly disagree
- Slightly agree
- Agree
- Strongly agree
- Don’t know

1. With regard to the infectious complication rate after prostate biopsy, I feel that in the institution where I am employed:*
   - On average there are few infectious complications
   - On average there are many infectious complications
   - Don’t know
   - Other (give further explanation)
2. With regard to the infectious complication rate after prostate biopsy, I feel that in the institution where I am employed:
   - There is a decreasing number of infectious complications after prostate biopsy
   - There is a increasing number of infectious complications after prostate biopsy
   - There is a stable number of infectious complications after prostate biopsy
   - Don’t know
3. In recent years, there has been an increase in the percentage of ciprofloxacin resistant Gram-negative bacteria. In urological patients this percentage has increased from 7% in 2000 to 19% in 2017. Were you aware of this increasing level of ciprofloxacin resistance?
   - Yes, I was aware of the increasing ciprofloxacin resistance to this extent
   - Yes, I was aware of the increasing ciprofloxacin resistance, but I had underestimated the extent of this
   - Yes, I was aware of the increasing ciprofloxacin resistance, but I had overestimated the extent of this
   - No, I wasn’t aware of the increasing ciprofloxacin resistance
4. Which of the following options would you consider as solution to the increasing percentage of ciprofloxacin resistant Gram-negative bacteria? (multiple answers possible)*
   - Empirical prophylaxis with ciprofloxacin remains my preference. If necessary, I accept a (somewhat) higher number of infectious complications after prostate biopsy.
   - Use of another antibiotic as empirical prophylaxis instead of ciprofloxacin.
   - Use of another antibiotic as empirical prophylaxis in addition to ciprofloxacin.
   - Rectal culture-guided antimicrobial prophylaxis strategy
   - Vaccination against infections caused by the bacterium *Escherichia coli*
   - Other
5. Which of the following options do you prefer as solution to the increasing percentage of ciprofloxacin resistant Gram-negative bacteria?
   - Empirical prophylaxis with ciprofloxacin remains my preference. If necessary, I accept a (somewhat) higher number of infectious complications after prostate biopsy.
   - Use of another antibiotic as empirical prophylaxis instead of ciprofloxacin.
   - Use of another antibiotic as empirical prophylaxis in addition to ciprofloxacin.
   - Rectal culture-guided antimicrobial prophylaxis strategy
   - Vaccination against infections caused by the bacterium *Escherichia coli*
   - Other
6. The Radboud university medical center (Radboudumc) is performing a prospective, multicenter study (PRO-SWAP) in which patients who undergo prostate biopsy based on randomization receive culture-guided antimicrobial prophylaxis or empirical prophylaxis with ciproflocacin.

Are you interested in participating in ths research? If you are interested, we will possibly approach you if we need more participating centers,.

- Yes
- No
- Perhaps
- Don’t know

**This is the end of this survey. Thank you for your participation!**

1. If you have any comments or observations, please let us know using the comments box below.

* Comment: question not interpretable due to methodological shortcomings.
