# Supplementary material for: Prostate biopsy techniques and pre-biopsy prophylactic measures: variation in current practice patterns in the Netherlands
Source: BMC Urol. 2020 Mar 12;20:24. doi: 10.1186/s12894-020-00592-8 (PMC7066741; doi:10.1186/s12894-020-00592-8)
Supplement: Supplementary file 2 — Additional file 2 Supplementary file II Antimicrobial prophylaxis regimens on hospital level and as specified by the (individual) respondents. The antimicrobial prophylaxis regimens as specified by the respondents on hospital and individual level are described. [file 12894_2020_592_MOESM2_ESM.docx]

| *Dose* | *Duration* | *Starting moment* | *n* | *%* |
| --- | --- | --- | --- | --- |
| **Ciprofloxacin** | | | **53** | **82.81** |
| 500 mg | Single dose | 0.5 hour before Bx | 1 | 1.69 |
|  |  | 1 hour before Bx | 16 | 25 |
|  |  | 1-2 hours before Bx | 2 | 3.13 |
|  |  | 2 hours before Bx | 8 | 12.50 |
|  | 1 day | 1 hour before Bx | 6 | 9.38 |
|  |  | 2 hours before Bx | 5 | 7.81 |
|  |  | Morning at the day of Bx and evening at the day of Bx | 2 | 3.13 |
|  | 1.5 day | Evening before, morning at the day of Bx and evening at the day of Bx | 3 | 4.69 |
|  | 3 days | Morning 1 day before Bx | 4 | 6.25 |
|  | 5 days | 48 hours before Bx | 1 | 1.69 |
| 750 mg | Single dose | 1 hour before Bx | 1 | 1.69 |
|  |  | 2 hours before Bx | 1 | 1.69 |
| 1000 mg | Single dose | 1 hour before Bx | 1 | 1.69 |
|  |  | 2 hours before Bx | 1 | 1.69 |
|  |  | 3 hours before Bx | 1 | 1.69 |
| **Levofloxacin** | | | **6** | **9.38** |
| 500 mg | Single dose | 1 hour before Bx | 3 | 4.69 |
|  |  | 2 hours before Bx | 1 | 1.56 |
|  | 1.5 day | Evening before and morning at the day of Bx | 1 | 1.56 |
|  | 3 days | Morning 1 day before Bx | 1 | 1.56 |
| **Ofloxacin** | | | **3** | **4.69** |
| 400 mg | Single dose | 1 hour before Bx | 1 | 1.56 |
|  | 3 days | Morning 1 day before Bx | 2 | 3.13 |
| **Trimethoprim/sulfamethoxazole** | | | **1** | **1.56** |
| 160/800 mg | 1.5 day | Evening before and morning at the day of Bx | 1 | 1.56 |
| **Ciprofloxacin + Fosfomycin** | | | **1** | **1.56** |
| 500 mg / 3 gram | Single dose | 2 hours before Bx | 1 | 1.56 |

***Antimicrobial prophylaxis regimens on hospital level***

***Antimicrobial prophylaxis regimens as specified by the (individual) respondents***

| *Dose* | *Duration* | *Starting moment* | *n* | *%* |
| --- | --- | --- | --- | --- |
| **Ciprofloxacin** | | | **131** | **83.97** |
| 250 mg | Single dose | 1 hour before Bx | 1 | 0.64 |
| 500 mg | Single dose | 0.5 hour before Bx | 1 | 0.64 |
|  |  | 0.5 – 1 hour before Bx | 1 | 0.64 |
|  |  | 1 hour before Bx | 25 | 16.03 |
|  |  | 1-2 hours before Bx | 2 | 1.28 |
|  |  | 2 hours before Bx | 27 | 17.31 |
|  |  | 3 hours before Bx | 1 | 0.64 |
|  | 1 day | 1 hour before Bx | 10 | 6.41 |
|  |  | 1-1.5 hours before Bx | 1 | 0.64 |
|  |  | 1-2 hours before Bx | 2 | 1.28 |
|  |  | 2 hours before Bx | 12 | 7.69 |
|  |  | 4 hours before Bx | 1 | 0.64 |
|  |  | Morning at the day of Bx and evening at the day of Bx | 2 | 1.28 |
|  |  | 12 hours and 1 hour before Bx | 1 | 0.64 |
|  | 1.5 day | Evening before, morning at the day of Bx and evening at the day of Bx | 8 | 5.13 |
|  | 3 days | 1 hour before Bx | 1 | 0.64 |
|  |  | Morning 1 day before Bx | 16 | 10.26 |
|  | 1-5 days | 1 hour before Bx | 1 | 0.64 |
|  | 5 days | 48 hours before Bx | 1 | 0.64 |
| 750 mg | Single dose | 1 hour before Bx | 3 | 1.92 |
|  |  | 2 hours before Bx | 6 | 3.85 |
|  | 3-5 days | 24 hours before Bx | 1 | 0.64 |
| 1000 mg | Single dose | 2 hours before Bx | 2 | 1.28 |
|  | 1 day | 1 hour before Bx | 3 | 1.92 |
|  |  | 2 hours before Bx | 1 | 0.64 |
|  |  | 4 hours before Bx | 1 | 0.64 |
| **Levofloxacin** | | | **9** | **5.77** |
| 500 mg | Single dose | 1 hour before Bx | 3 | 1.92 |
|  |  | 2 hours before Bx | 3 | 1.92 |
|  | 1 day | 24 hours before Bx | 1 | 0.64 |
|  | 1.5 day | Evening before and morning at the day of Bx | 1 | 0.64 |
|  | 3 days | Morning 1 day before Bx | 1 | 0.64 |
| **Ofloxacin** | | | **5** | **3.21** |
| 400 mg | Single dose | 1 hour before Bx | 2 | 1.28 |
|  | 3 days | Morning 1 day before Bx | 3 | 1.92 |
| **Trimethoprim/sulfamethoxazole** | | | **7** | **4.49** |
| 160/800 mg | 1 day | 1 hour before Bx | 2 | 1.28 |
|  |  | 1.5 hours before Bx | 1 | 0.64 |
|  |  | 2 hours before Bx | 2 | 1.28 |
|  |  | 12-18 hours before Bx | 1 | 0.64 |
|  |  | Evening before and morning at the day of Bx | 1 | 0.64 |
| **Cefazolin** | | | **1** | **0.64** |
| 500 mg | Single dose | 1 hour before biopsy | 1 | 0.64 |
| **Ciprofloxacin + Fosfomycin** | | | **3** | **1.92** |
| 500 mg / 3 gram | Single dose | 1 hour before Bx | 2 | 1.28 |
|  |  | 2 hours before Bx | 1 | 0.64 |
