# Supplementary material for: Prostate biopsy techniques and pre-biopsy prophylactic measures: variation in current practice patterns in the Netherlands
Source: BMC Urol. 2020 Mar 12;20:24. doi: 10.1186/s12894-020-00592-8 (PMC7066741; doi:10.1186/s12894-020-00592-8)
Supplement: Supplementary file 3 — Additional file 3 Supplementary file III Prior surveys on pre-prostate biopsy prophylaxis published since 2010. Schematic overview of surveys conducted by others since 2010 about prophylactic measures around transrectal prostate biopsy. [file 12894_2020_592_MOESM3_ESM.docx]

***Prior surveys on pre-prostate biopsy prophylaxis published since 2010***

| **Author** | **Year of publication** | **Country** | **Respondents (*n)*** | **Use of antimicrobial prophylaxis (%)** | **Choice of antimicrobial prophylaxis (%)** | | **Duration of treatment (%)** | | **Route of administration** | | **Measures of infection control (%)** | |
| --- | --- | --- | --- | --- | --- | --- | --- | --- | --- | --- | --- | --- |
| Boehm *et al.*(1) | 2018 | Germany | 455 | 98 | Single agent  Fluoroquinolones  Other (gentamicin,  fosfomycin,  ampicillin/sulbactam)  NA | 89.5  4.0  6.6 | Single dose  > 1 dose  Median:  4 days | 10.1  89.9 | NA | | Pre-interventional urine culture  Rectal swab  Antimicrobial lubricant | 44.6  23.9  33.1 |
| Johnson *et al.*(2) | 2015 | United States | 252 | NA | Single agent  Fluoroquinolones  Cephalosporin  Carbepenem  Aminoglycoside  Cefazolin  Combination  Fluoroquinolone +  aminoglycoside  Other combination  Variable | 47.6  4.8  1.2  0.8  0.4  8.3  25.0  11.9 | Single dose  Several doses ≤ 24 h  1 – 2 days  ≥ 3 days | 55.6  21.5  17.3  5.6 | NA | | Rectal swab | 9 |
| Davis *et al.(*3) | 2014 | Australia and New Zealand | 150 | 98.6 | Mostly fluoroquinolones  Carbepenem IV in  patients at risk of FQ  resistance | 27.7 | 1 day  3 days  5 days | 22.3  56.8  11.5 | IV 69.4  At least sometimes | | Rectal swab  Rectal swab in patients at risk  Standard enema  Antiseptic enema | 2  9.5  29.9  10.8 |
| Carlsson *et al.*(4) | 2012 | Sweden | 137 | 100 | Single agent  Ciprofloxacin  Co-trimoxazole  Norfloxacin  Ciprfloxacin or  Co-trimoxazole | 83.8  11.8  0.7  3.7 | Single dose at time of biopsy  Single dose  1-2 h before biopsy  1 day  2-3 days  ≥ 4 days | 63.5  4.4  11.7  17.6  2.9 | NA | | NA | |
| Hillelsohn *et al.(*5) | 2012 | United States | 679 | NA | Single agent  Fluoroquinolone  Other  Combination  Fluoroquinolone +  Gentamycin IM  Fluoroquinolone +  Cephalosporin IM  Fluoroquinolone oral +  IV  Other | 65.7  6.4  16.6  4.9  1.6  4.9 | 1 day  2 days  3 days  ≥ 4 days | 59  19  14  8 | Oral  IM  IV  Oral + IM  Oral + IV | 69.8  1.4  0.7  24.4  3.6 | Enema | 97 |
| Smyth *et al.(*6) | 2012 | Ireland | 29 hospitals | 100 | Single agent  Ciprofloxacin  Gentamycin  Metronidazole  Ofloxacin  Co-Amoxiclav | 46  23  14  12  5 | Single dose  1 day  2 days  3 days  4 days  5 days  10 days | 14  29  11  18  11  14  3 | Oral  IM  IV  Oral + IM  Oral + IV  Oral + IV + per rectum | 62  0  7  0  28  3 | NA | |

**References**

1. Boehm K, Siegel FP, Schneidewind L, Kranz J, Spachmann P, Frank T, et al. Antibiotic Prophylaxis in Prostate Biopsies: Contemporary Practice Patterns in Germany. Front Surg. 2018;5:2.

2. Johnson JR, Polgreen PM, Beekmann SE. Transrectal prostate biopsy-associated prophylaxis and infectious complications: report of a query to the emerging infections network of the infectious diseases society of america. Open Forum Infect Dis. 2015;2(1):ofv002.

3. Davis P, Paul E, Grummet J. Current practice of prostate biopsy in Australia and New Zealand: A survey. Urol Ann. 2015;7(3):315-9.

4. Carlsson S, Bratt O, Stattin P, Egevad L. Current routines for transrectal ultrasound-guided prostate biopsy: a web-based survey by the Swedish Urology Network. Scand J Urol Nephrol. 2012;46(6):405-10.

5. Hillelsohn JH, Duty B, Blute ML, Jr., Okhunov Z, Kashan M, Moldwin R, et al. Variability of transrectal ultrasound-guided prostate biopsy prophylactic measures. Can J Urol. 2012;19(6):6573-7.

6. Smyth LG, Mulvin DW. Antibiotic prophylaxis for transrectal ultrasound biopsy of the prostate in Ireland. Ir J Med Sci. 2012;181(1):33-5.
